# Providing community-based health practitioners with timely and accurate discharge medicines information

**DOI:** 10.1186/1472-6963-12-453

**Published:** 2012-12-10

**Authors:** Alice V Gilbert, Bhavini Patel, Melanie Morrow, Desmond Williams, Michael S Roberts, Andrew L Gilbert

**Affiliations:** 1 Pharmacy Department, Royal Darwin Hospital, Rocklands Drive, Darwin 0810, Australia; 2 School of Pharmacy and Medical Science, University of South Australia, North Terrace, Adelaide, Australia; 3 School of Medicine, Queensland University, Sir Fred Schonell Drive, Queensland, 4072, Australia

**Keywords:** Continuum of medication management, Discharge medicines, Clinical handover

## Abstract

**Background:**

Accurate and timely medication information at the point of discharge is essential for continuity of care. There are scarce data on the clinical significance if poor quality medicines information is passed to the next episode of care. This study aimed to compare the number and clinical significance of medication errors and omission in discharge medicines information, and the timeliness of delivery of this information to community-based health practitioners, between the existing Hospital Discharge Summary (HDS) and a pharmacist prepared Medicines Information Transfer Fax (MITF).

**Method:**

The study used a sample of 80 hospital patients who were at high risk of medication misadventure, and who had a MITF completed in the study period June – October 2009 at a tertiary referral hospital. The medicines information in participating patients’ MITFs was validated against their Discharge Prescriptions (DP). Medicines information in each patient’s HDS was then compared with their validated MITF. An expert clinical panel reviewed identified medication errors and omissions to determine their clinical significance. The time between patient discharge and the dispatching of the MITF and the HDS to each patient’s community-based practitioners was calculated from hospital records.

**Results:**

DPs for 77 of the 80 patients were available for comparison with their MITFs. Medicines information in 71 (92%) of the MITFs matched that of the DP. Comparison of the HDS against the MITF revealed that no HDS was prepared for 16 (21%) patients. Of the remaining 61 patients; 33 (54%), had required medications omitted and 38 (62%) had medication errors in their HDS. The Clinical Panel rated the significance of errors or omissions for 70 patients (16 with no HDS prepared and 54 who’s HDS was inconsistent with the validated MITF). In 17 patients the error or omission was rated as insignificant to minor; 23 minor to moderate; 24 moderate to major and 6 major to catastrophic. 28 (35%) patients had their HDS dispatched to their community-based practitioners within 48 hours post discharge compared to 80 (100%) of MITFs.

**Conclusion:**

The MITF is an effective approach for the timely delivery of accurate discharge medicines information to community-based practitioners responsible for the patient’s ongoing care.

## Background

Accurate and timely information on medication management along the continuum of care is essential to achieving best outcomes for patients and for avoiding harm [1-4]. Approximately 3% of hospital admissions occur due to medication-related problems [5]. Often these problems are due to miscommunication regarding the continuation/discontinuation of medications on hospital discharge [5]. The interface between hospital and the community care is a high risk area for medication misadventure [5-7].

The Australian Pharmaceutical Advisory Council (APAC), a Federal Government appointed, multidisciplinary council, has attempted to address these issues by preparing Guiding Principles for Achieving the Continuum of Medication Management [8]. The Guiding Principles state that “Health service managers and health care professionals are jointly and individually accountable for making sure that activities to support the continuity of medication management are implemented.” (8, page 17). The Guiding Principles also reinforce the responsibility of health professionals to work in partnership with patients in all aspects of medication management [8]. Similarly, the Society of Hospital Pharmacists of Australia’s (SHPA) Standards of Practice for the provision of medication reconciliation require that “when a patient is transferred to another episode of care the transferring health professional should supply comprehensive, complete and accurate information to the health care provider responsible for continuing the consumer’s medication management.” ([9], page 2). Continuity of care between hospitals and the community sector is a key element in Australia’s Health Care Reforms [10], and the Federal Government has provided extra funding for hospitals conditional on implementation of the Guiding Principles.

The need for high quality clinical handover is exaggerated in the Northern Territory (NT) of Australia: It covers a large geographic area and it has a relatively small, transient, widely distributed and remote population (n = 225900): 30% of the NT population is Aboriginal and Torres Strait Islanders (ATSI)^a^. The life expectancy for male ATSI is 61.5 years, 17 years less than non-indigenous males, and for female ATSI 69.2 years, 13 years less than non-indigenous females. The median age at death of ATSI males is 50 years. [11] Many issues, including low health literacy, [12] differing cultural beliefs about health and illness, access to health services and availability of health professionals limit people’s ability to access and navigate their way through the complex health system. The majority of health services are accessed through health centres based in remote communities. The case mix at the study hospital differs from other large Australian hospitals; patients are younger and presentations for trauma and severe sepsis are higher. The Emergency Department sees approximately 60000 patients per year, ATSI account for 30% of attendance and 50% of admissions. It should be noted that in this study, patients at high risk of medication misadventure were selected. Nearly half of those selected were ATSI and their average age was 58 years; 15 years younger than the rest of the study cohort (See Table 1).

**Table 1:** Patient characteristics

	**Aboriginal and Torres strait islanders (n = 37)**	**Non-Aboriginal and Torres strait islanders (n = 43)**	**All patients (n = 80)**
Mean age (years), (Range)	58 (33–78)	73 (29–97)	66.1 (29–97)
Gender (Female), n (%)	16 (43%)	19 (44%)	17.6 (43.5%)
Mean number of Medical Conditions (range)	7.4 (3-18)	7.3 (2-12)	7.3 (2-18)
Mean number of medication on discharge (range)	8.6 (3-16)	9.4 (3–23)	9.1 (3–23)
Mean number of changes on Discharge (range)	3 (0–8)	4 (1-13)	3.5 (0–13)

The current discharge procedure at the study hospital requires an intern or registered medical officer to complete an electronic Hospital Discharge Summary (HDS). The HDS contains a section documenting changes to medications and provides a list of discharge and continuing medicines. The hospital provides the HDS to the patient’s general medical practitioner (GP), aged-care facilities, remote area clinics, or the patient once the HDS has been completed.

Evidence indicates that a patient in an urban area will visit their pharmacy within 10 days and their doctor 30 days after discharge [13]. While most GPs are provided with a discharge summary, community pharmacists are rarely provided with this information and patients are not encouraged to notify their community-based practitioners of recent hospitalisations and changes to their medicines. [13] In this study, the MITF was delivered to medical practitioners, community pharmacists and remote area clinic staff.

Clinical pharmacists from the study hospital had reported frequent contacts from community-based practitioners seeking clarification of the medicines information provided in the HDS. They also identified that the length of time for the provision of the HDS to the patient’s community-based practitioners was outside of hospital guidelines of 48 hours. In response to this information, pharmacists at the study hospital developed a Medicines Information Transfer Fax (MITF).

This study compares the number and clinical significance of medication errors and omissions in, and the timeliness of delivery of, discharge medicines information provided to community-based health professionals in the existing Hospital Discharge Summary (HDS) and the Medicines Information Transfer Fax (MITF).

## Method

The study was conducted using a convenience sample of 80 MITF’s prepared for patients at high risk of medication misadventure during a four month period June 2009 – October 2009. At the time the study was conducted there were 9 clinical pharmacists employed in the hospital and 1000 discharges/week. The clinical pharmacists selected patients for whom they developed a MITF based on their assessment of those most at risk of medication misadventure post discharge. On average 30 MITFs were completed each week. The project team did not have had access to all MITF’s and DPs completed within the study period because of filing systems adopted by individual pharmacists; for example some filed their MITF’s on a monthly basis, and the hospital, for example storing DP off-site as part of their record archiving process. The sample of 80 represented 20% of available MIFTs and was considered sufficient to identify significant differences, if they existed, between the current HDS and the MITF.

High risk patients were defined as; those taking multiple medications, those who had medication changes during admission, those suspected of poor adherence, those taking high risk or complex medications and those with multiple medical conditions. They had a medication history and reconciliation conducted for the admission and at the point of discharge as part of standard care. The reconciled Discharge Prescription (DP) was used to complete a MITF which also included a complete list of discharge medications and provided information on any medication-related changes that occurred during the admission. MITFs were faxed to the patient’s GP or Remote Medical Officer and community pharmacist at the time of the patients discharge. The date of sending the MITF was recorded on the faxed document and filed in the pharmacy department. The aim was to have the MITF delivered to the patient’s community-based practitioners within 48 hours of discharge. The time to delivery of the HDS was calculated using the study hospital’s admission and discharge software.

**Figure 1:**
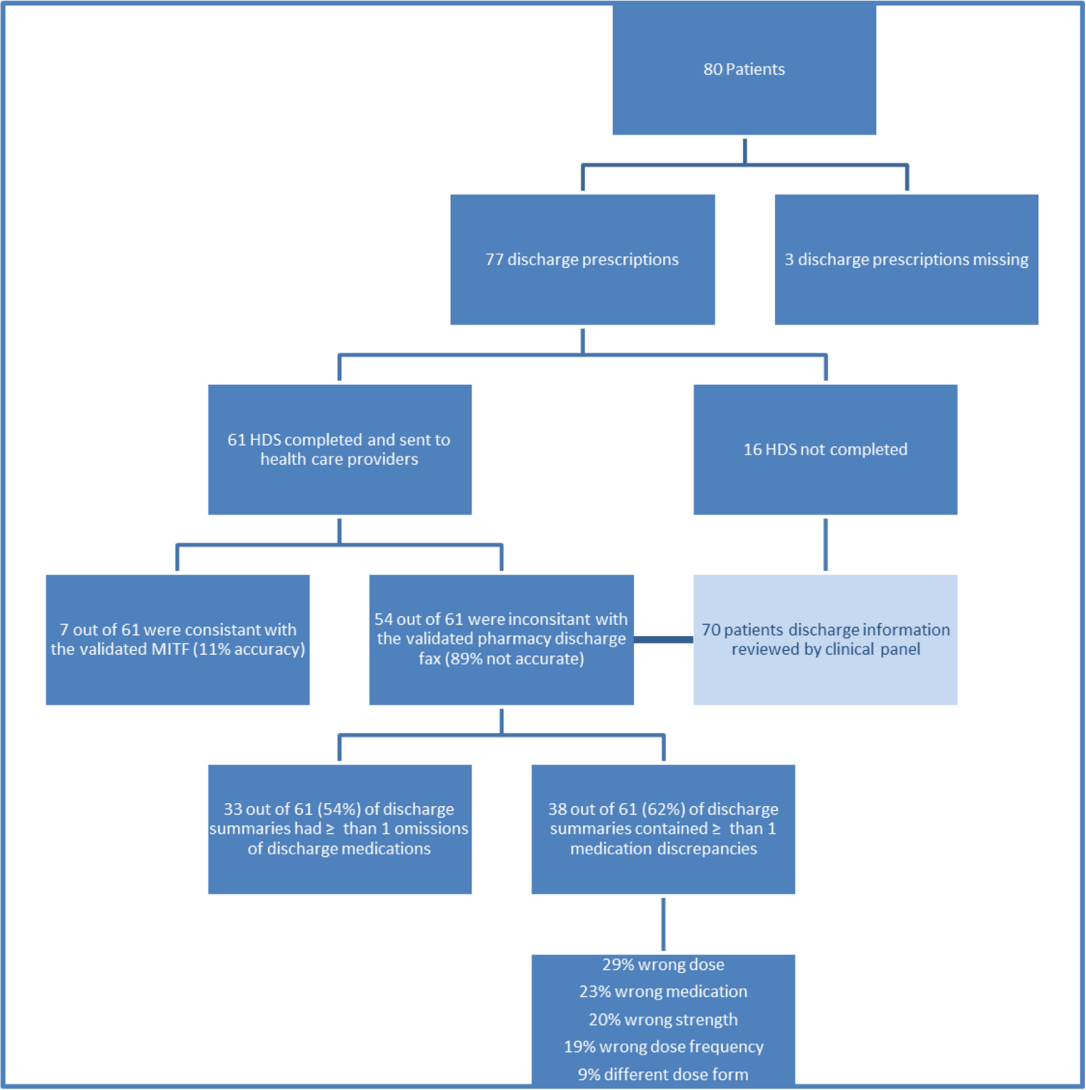
Audit flow chart.

Two members of the project team audited the MITF against the gold standard, the DP. The DP was considered gold standard as it had been reconciled by a clinical pharmacist as part of the routine hospital medicines reconciliation process. Medicines information contained in the HDS was compared with that in the validated MITF. Medication errors in the HDS were categorised into five classes; wrong dose, wrong medication, wrong strength, wrong dose frequency or wrong dose form. Omissions were also documented (Figure 1: Audit flow chart).

Medication errors and omissions identified in the audits were reviewed for their clinical significance by a panel of three senior clinical pharmacists. Panel members were blinded to the patient’s name and the name of the pharmacist who had prepared the MITF. Clinical significance was determined from information on the patient’s health status and the accuracy and quality of information provided at discharge. Prior to the review advice was sought by the panel from the hospital’s liaison GP to gain insight into how a community-based prescriber would interpret the medication information. For example if medications were omitted from the HDS, the advice given to the panel was that a medical practitioner would interpret this as there being no change to current medication and would continue the patient’s pre-hospitalisation medicines, unless deemed clinically inappropriate. Each panel member rated the clinical significance of each error or omission independently using the “consequence or impact scale” of the SHPA assessment of intervention tool [14].The five point Likert scale ranged from 1 (insignificant), 2 (minor), 3 (moderate), 4 (major) to 5 (catastrophic). The overall agreement between the three assessors was measured using the Kappa statistic [15].

**Table 2:** Examples from data set of classification category for errors and omissions

**Consequence and impact ratings**	**EXAMPLES**
Insignificant to minor	84 year old non-indigenous male discharged after treatment for community acquired pneumonia. Past medical history includes; fractured neck of femur, urinary incontinence, chronic cardiac failure, atrial fibrillation, and gastro oesophageal reflux disease. Changes to medication during admission included commencing digoxin, and ceasing Bisoprolol, spironolactone, and ramipril.
	Pharmacist had incorrect dosing for omeprazole on MITF. Omeprazole 40 mg nocte on discharge prescription and 20 mg BD in the MITF all other medication information was transferred correctly on MITF. Insignificant risk to patient.
Minor to moderate	75 year old indigenous male discharged after being admitted for shortness of breath due to worsening chronic cardiac failure. Past medical history includes; chronic obstructive airways disease (current smoker), atrial fibrillation, chronic cardiac failure, myocardial infarction and hypertension. During admission frusemide was increased from 40 mg mane to 60 mg mane.
	All medications were omitted in the HDS, therefore from advice from the hospital’s liaison GP assumed all medications and doses were continued. Primary health care professional would not be aware of increase in frusemide dose therefore minor risk to patient of deterioration and potential re-admission.
Moderate to major	55 year old non-indigenous male discharged post an episode of chest pain on the back ground of high risk cardiac disease. Past medical history includes; ST segment elevation myocardial infarction, severe arterial stenosis (ischaemic heart disease), asthma, and transient ischaemic attack. Patient was discharged after an increase in nicorandil from 5 mg BD to 10 mg BD and an increase in Isosorbide mononitrate from 60 mg mane to 90 mg mane.
	This patient did not have HDS completed. Therefore is a moderate risk that this patient will be re-admitted due to reverting back to original dosing and increasing angina attacks. Also risk of confusion of medications to patient.
Major to catastrophic	37 year old indigenous female discharged with osteomyelitis. Past medical history of end stage renal disease (on dialysis), chronic intermittent vomiting since starting dialysis which has been fully investigated, type 2 diabetes, hypertension, dyslipidaemia, and a previous right toe amputation with residual osteomyelitis.
	During admission diabetic control was reviewed and changed by increasing insulin dose from 10 units nocte to 14 units nocte and metformin XR 500 mg mane was ceased.
	Osteomylitis treatment was changed by ceasing Trimethoprim/ Sulphamethoxazole 160/800 mg BD and folic acid 5 mg and commencing on Doxycycline 100 mg mane and ciprofloxacin 500 mg nocte for lifelong treatment.
	Metformin XR 500 mg was included in HDS (it had been ceased during admission) with new dose of insulin correct, therefore putting patient at risk of hypoglycaemia.
	Doxycycline and ciprofloxacin had been omitted from HDS therefore primary health care provider would not be aware of change to antibiotic regime. High risk of further amputations and losing foot due to osteomyelitis infection.

## Results

Of the 80 patients whose MITFs were selected for this study, three patient’s did not have a DPs available for comparison. Of the 77 MITFs with DPs available: 6 (8%) MITFs had errors; Four errors were rated by the clinical panel as “insignificant to minor” and two errors were viewed as “minor to moderate”.

A completed HDS was available for 61 (79%) of the 80 patients within the audit time frame; these were compared with the validated MITFs. Seven of the 61 (11%) HDS’s were consistent with the MITF; 149 medication omissions and 100 errors were identified in the remaining 54 HDS’s. An omission occurred when a medication appeared on the discharge prescription but was not documented in the HDS. Errors were categorised into five classes; wrong dose (29, 29%), wrong medication (23, 23%), wrong strength (20, 20%), wrong dose frequency (19, 19%) or wrong dose form (9, 9%).

The clinical panel members independently rated the clinical significance for the 70 patients who had errors or omissions in their HDS’s or had no HDS completed. Based on advice from the hospital’s liaison GP, patients with no HDS completed were reviewed for their clinical risk associated with no information being provided at handover. Of the 70 reviews; for 17 (24%) patient’s the clinical significance was “insignificant to minor”; 23 (33%) “minor to moderate”; 24 (34%) “moderate to major” and 6 (9%) were “major to catastrophic” (See Table 2).

Analysis of the individual panel member’s ratings of clinical significance indicated a satisfactory level of inter-rater agreement (Kappa for overall agreement = 0.7).

The HDS was dispatched to 28 (35%) patient’s health-care providers within the study hospital’s guideline of 48 hours; 38 (47%) were sent within seven days and 55 (69%) were sent within 30 days: 25 (31%) HDSs had not been dispatched after 30 days post discharge. All MITFs were dispatched within 48 hours post-discharge.

## Discussion

Despite national guidelines, extensive research, the availability of funding for more pharmacists to implement the APAC guiding principles for the continuum of care and professional practice standards related to clinical handover, medication management along the continuum of care is still problematic. This audit of the current discharge process has found significant issues in terms of the accuracy, quality and timeliness of delivery of the HDS to community-based practitioners responsible for the next episode of care. Studies from Europe, North America and New Zealand have produced similar results [1-4].

Our analysis of the clinical significance of medication errors or omissions in the HDS, based on information on the patient’s health status, and the accuracy and quality of information provided at discharge revealed significant risks to patient’s well-being. An alarming 43% of medication errors or omissions discovered in our review on 80 HDS were rated as moderate to catastrophic.

While our findings are also consistent with previous Australian studies [5,6,16], the context in which these errors and omissions occur adds extra cause for concern. Nearly 50% of patients in this audit were Aboriginal or Torres Strait Islanders (ATSI) and clinical handover was often to remote area health services. The lack of a discharge summary, or errors and omissions in the discharge medicines information provided, may be even more significant because of poorer health status, low health literacy [12] and the low access to medical practitioners and community pharmacy services [17,18]. Many adult ATSI patients have multiple chronic health problems at a much younger age than non-indigenous people. Over 35% will have diabetes, often coupled with other chronic conditions such as mental health problems, arthritis and cardiovascular disease [11,19]. Medicines management is consequently complex, with multiple medications in use.

The study hospital’s current policy for discharging patients with only 7 days of medications, with an option of an increased supply for patients returning to a remote community, may exacerbate many of the problems identified in this audit. This may have significant health consequences, when people with multiple chronic health problems visit their community pharmacist, health clinic or medical practitioner, if accurate information on medication changes associated with the hospitalisation are not available within this timeframe. Findings of this study indicate that only 47% of HDSs were delivered within 7 days from discharge.

Due to work load and time restraints the clinical pharmacists only focused on high risk patients. The ability of the clinical pharmacists’ to deliver an accurate and high quality medication discharge process, with timely delivery of the medicines discharge information to those health practitioners involved in the next episode of care was demonstrated in this project.

The time is right for a new model for the hospital discharge process. Guidance for hospital and community pharmacists to support patient discharge from hospital has recently been published by the United Kingdom National Prescribing Centre [20]. The guidance aims to foster relationships between community pharmacists and hospital staff. In Australia, new funding is expected to be available for hospital pharmacists, GP’s and community pharmacists to deliver a comprehensive medicines discharge process, including a Hospital Initiated Home Medicines Review [21]. Developing the relationships between community pharmacists, hospital pharmacists, medical practitioners, health clinic staff and engaging patients, will be critical success factors.

Limitations were identified while conducting this study. Pharmacists prioritised patients with complex medication-related issues for a MITF. These patients have many, and often “last minute” changes to their medications near discharge, making it difficult for those completing the HDS to accurately complete the discharge medication record. Further, medical practitioners in the hospital were not asked about how they make decisions about information to be included in their HDS. They may, for example, concentrate more on accurate recording of diagnoses, expecting pharmacists and nurses to ensure accurate recording of medication changes. Whilst the hospital’s liaison GP was asked for assistance when the clinical panel was rating the significance of omitted medicines, not having prescribers represented on the panel was a limitation that should be rectified in subsequent audits. The usefulness of information contained in the MITF has not been formally tested by requesting feedback from the community-based practitioners. There is the likelihood of confusion if a community-based practitioner received conflicting information from the hospital, as may be the case when the HDS and the MIFT have different medication management information. This may increase time demands on the community-based practitioners as they will need to contact the hospital to clarify discrepancies. Finally the small sample size was determined by the capacity of the research team to undertake this study while maintaining a full-time case load and other professional responsibilities. The magnitude of the issues uncovered, even with this small sample size, has meant however meaningful conclusions can be drawn.

Notwithstanding these limitations, this simple innovation has identified significant issues with the existing HDS and has demonstrated that the MITF delivered accurate, timely and complete handover of medication management information from the hospital service providers to community-based practitioners. The study was led by clinical pharmacists working full-time in the hospital. Its success is an important example of the usefulness of imbedded practitioner research methods [22]. Being imbedded in the setting means that the researchers knew from conversations about the limitations of the HDS, the frustrations caused to community–based practitioners and the risk to patients when they had no discharge information or when the information provided was not correct. The researchers also knew about the hospitals culture and what was possible in terms of engagement of other health workers in the study, work force capacity and pathways to implement change based on the study results.

The promise of an information technology (IT) solution to the problem of timely and accurate clinical handover has not yet been realised. In the NT alone there are at least five different IT patient administration systems (for example primary care information systems, remote area information systems, GP prescribing software and pharmacy dispensing software) that need to be negotiated in what is a highly transient population group. The MITF provides an ideal interim measure for consideration as electronic support for discharge processes are implemented.

The results reported in this paper relate only to the introduction period of the MITF in 2009. Based on the data generated in the study, the MITFs are now required to be completed by all pharmacists as part of their daily duties. The MITF template has also been adopted by four of the five hospitals in the NT (the remaining hospital does not have a pharmacist). Training in the completion of the MITF is now also fully integrated into the orientation process for new pharmacists to the NT hospital Network. The MITF template is currently being mapped by the programmers of the electronic based hospital discharge software to provide the capability for prescribers to examine and utilise the information available in the MIFT at the time of creating their HDS.

## Conclusion

This study demonstrated increased accuracy, quality and timeliness of medication information relayed from the hospital to community-based practitioners through the use of the MITF. The MIFT provides an excellent platform for further strengthening and standardising the medicines discharge processes in hospitals. Based on the results of this study the MITF is now a formal requirement of the hospital’s patient discharge processes.

## Endnote

^a^ Torres Strait Islanders are the indigenous people of the Torres Strait Islands, which are a part of Australia. They are culturally and genetically linked to Melanesian and Papua New Guinea peoples, and are regarded as being distinct from other Aboriginal peoples from the rest of Australia.

## Abbreviations

APAC: The australian pharmaceutical advisory council; ATSI: Aboriginal and torres strait islanders; DP: Discharge prescriptions; GP: General medical practitioner; HDS: Hospital discharge summary; IT: Information technology; MITF: Medicines information transfer fax; NT: Northern territory; SHPA: Society of hospital pharmacists of Australia’s.

## Competing interests

The authors declare that they have no competing interests.

## Authors' contributions

AVG Leader of the research team and undertook collection of all data, analysis of all data, interpretation of data and writing first draft of the paper and finalising paper for submission to journal. BP Facilitated access to data, assisted with analysis of data, revised first draft of paper and finalisation of paper for submission. Assisted in design of the Medicines Information Transfer Fax. MM Facilitated access to data, assisted with analysis of data, revised first draft of paper and finalisation of paper for submission. Assisted in design of the Medicines Information Transfer Fax. DW Participated in preparation of first draft of the article and subsequent reviews of the article. MR Guided conceptual frame work for the study and participated in reviews of the article. ALG Guided conceptual frame work for the study and participated in reviews of the article. All authors read and approve the final manuscript.

## Pre-publication history

The pre-publication history for this paper can be accessed here:

http://www.biomedcentral.com/1472-6963/12/453/prepub
